# State of the Globe: *Helicobacter pylori* and Hepatitis C Together Hamper Health

**DOI:** 10.4103/0974-777X.59243

**Published:** 2010

**Authors:** Mamun-Al-Mahtab

**Affiliations:** *Department of Hepatology, Bangabandhu Sheikh Mujib Medical University, Viral Hepatitis Foundation, Bangladesh*

*H. pylori* and hepatitis C virus (HCV) are respectively the leading bacterial and viral[1] human disease aetiologies globally. Around half of the world's population is infected with *H. pylori.*[2] The incidence rises steadily with age. In the UK, approximately 50% population (over 50 years of age) is infected with *H. pylori*. In the developing world, this figure may be as high as 90%. Vast majority of the infected population, however, remains asymptomatic. Around 90% patients with duodenal ulcer and 70% with gastric ulcer are infected with these bacteria.

*H. pylori* is noninvasive. It produces chronic gastritis by provoking local inflammatory response in the epithelium through release of a range of cytokines. The organism's motility allows it to colonize deep beneath the mucosa. It exclusively colonizes gastric mucosa and is found in duodenum only in association with gastric metaplasia.

The hepatitis C virus (HCV) was first discovered in 1989 by Michael Houghton and colleagues at Chiron. It was rapidly recognized that the new virus was responsible for the majority of cases of non-A, non-B hepatitis. Subsequent studies have shown that HCV is a single stranded RNA virus and has a genetic organization that is similar to the yellow fever virus (a flaviivirdae).

HCV infects approximately 170 million population worldwide.[3] Chronic hepatitis C (CHC) is a major public health problem and a leading cause of chronic liver disease globally including in our region, especially in Pakistan, where the prevalence of the virus is over 8%. Although the prevalence of hepatitis C virus (HCV) in Bangladesh and India is less than 1%, it represents a very large HCV reservoir, posing significant threat to the health and economy of these upcoming nations.[4]

There are a number of ways HCV can be transmitted. A study of 16,400 patients, attending an outpatient department of a district hospital in Buner, Pakistan from January 1998 to December 2002, was carried out to look at modes of transmission. It found that, those who were infected with HCV, had a history of some form of injection use, 6.92% had some form of major surgery, 1.06% had a blood transfusion, 9.72% had a dental procedure, 0.39% had some tattooing done, and 44.20% had been shaved by community barber (see below). Unfortunately, the lack of data in a control population makes interpretation of these data problematic, although it is clear that any procedure that exposes patients to blood provides a common route for infection.[5]

HCV is primarily transmitted through infected blood or blood products. Injectable drug abuse remains an important mode of transmission of the virus including in the US. Other potential sources of HCV transmission include exposure to infected blood and sexual exposure. Sharing of razor, toothbrush, etc. also can lead to HCV transmission. The virus is not transmitted by hugging and sharing of eating utensils, dresses or toilets. Patients with hemophilia and those on renal dialysis should be tested for HCV. Other situations that deserve to be considered include traditional medical practices like acupuncture, body piercing, tattooing and commercial barbering.

HCV replicates in the hepatocyte cytoplasm and it is not clear whether liver cell damage is due to direct viral cytopathic effects or to immune mediated liver cell damage. It is known that HCV has a very rapid replication cycle with an estimated 10^10^–10^12^ virions per day being produced. This rate of rapid replication leads to rapid viral mutation and genetic drift.

Infection with the HCV may lead to an acute, self limiting infection that is often asymptomatic. That said, such patients are uncommon with only 20% of infected patients presenting in this way and usually present with the incidental discovery of antibodies against hepatitis C. Such patients are HCV RNA negative by PCR testing and further follow up is not required. The vast majority of patients who are exposed to HCV develop a chronic persistent infection with continuing viraemia that may last for decades.

The natural history of chronic HCV infection is still not clear. It is known that significant liver injury is uncommon in patients whose disease duration is less than 20 years but prolonged infection often results in chronic liver disease, cirrhosis and hepatocellular carcinoma. The rate of progression of chronic HCV infection to fibrosis and then to cirrhosis is variable and is influenced by a number of factors such as concomitant consumption of excessive alcohol, acquiring the infection at an older age (>40 years), co-infection with HBV or HIV, degree of liver inflammation on biopsy, male gender, imunosuppression, insulin resistance, NASH (nonalcoholic steato-hepatitis), hemochromatosis and schistosomiasis. Of these factors male gender, excessive alcohol consumption and co-infection with HIV or HBV play the most significant roles in liver fibrosis progression.

The rate of progression of liver fibrosis in chronic HCV infection during the early years of infection is variable but is usually very slow and most patients do not develop significant scarring for at least 20 years. A minority of patients can develop rapidly progressive fibrosis leading to cirrhosis after a relatively short time lasting a few years. During the later stages of hepatitis C infection the rate of progression of fibrosis accelerates which can lead to the development of cirrhosis. Up to 15–20% have a risk of developing cirrhosis after 20 years of infection.

Once cirrhosis has developed complications can develop. An Italian study looking at a 17 year cohort of 214 patients with compensated cirrhosis due to chronic HCV found that after 114 months of follow up HCC had developed in 68 individuals (32%) which was the main cause of death; ascites had formed in 50 (23%); jaundice in 36 (17%); upper GI bleeding in 13 (6%); and hepatic encephalopathy in 2 (1%). The clinical status remained unchanged in 154 (72%) but progression to Child-Pugh class B occurred in 45 (21%) and class C in 15 (7%).The cumulative probability of events such as HCC, ascities, jaundice, GI hemorrhage and portal-systemic encephalopathy all increased with time.[6–8]

*H. pylori,* on the other hand, leads to acute and chronic gastritis and duodenal and gastric ulcers.[9,10] Moreover, it can result in mucosa-associated lymphoid tissue (MALT) lymphoma.[11] It is particularly associated with the development of low grade lymphoma and superficial gastric lymphomas may be cured by *H. pylori* eradication.

The organism is also a recognized cause of gastric adenocarcinoma.[12] It produces mutagnic nitrites from dietary nitrates. Chronic inflammation with generation of recative oxygen species and depletion of normally abundant anti-oxidant ascorbic acid are also important. *H. pylori* infected individuals usually have normal or increased acid secretion. A few however become hypo or achlorhydric and are thought to be at greatest risk. It is estimated that *H. pylori* is responsible for 60–70% cases of gastric carcinoma and acquisition of the bacteria at an early age is associated with increased risk.

Although there is some evidence of *H. pylori* induced hepatotoxicity *in vitro*[13] and in animal models, it is yet to be established. High prevalence of *H. pylori* is, however, well-documented in patients with chronic liver disease (CLD).[14,15] And contrary to our initial belief that *H. pylori* is sensitive to bile, we now know that this pathogen is detectable[16,17] and can also survive is bile rich environment.[18,19]

*H. pylori* DNA has been isolated from liver of patients with CLD and HCC[20] resulting from other established aetiology. This has led to the postulation of a hypothesis that *H. pylori* may well be involved in the pathogenesis of CLD and specially HCC. This is especially because some studies, including the one published in this issue, has demonstrated that there is increasing association between *H. pylori* infection with increasing Child-Pough and MELD scores in CLD.[15]

Chronic hepatitis is an inflammatory process and as with any other chronic inflammation, it too is characterized by a rise in pro-inflammatory cytokines like IL1, IL6, TNF, etc.[21] Viruses, including HCV, are capable of inducing limited inflammation,[22] contrary to bacteria like *H. pylori,* which are potent inducers of the inflammatory cascade.[23] It has been shown that *H. pylori* can cause proto-oncogene activation that is likely to the key step in the pathway of *H. pylori* induced neoplasia.[24,25]

The currently available information is too sparse to point to *H. pylori*, as having any definite role in the evolution of CLD and/or HCC. But *H. pylori* eradication therapy has shown to be beneficial in patients with CLD and/or HCC who are co-infected with *H. pylori*. It is clear that there is increasing association of *H. pylori* with CLD. Moreover, both *H. pylori* and HCV have pathogenic influence on gastric and liver epithelium, including inducing the risk of malignant transformation. This definitely warrants further studies. An article from an Egyptian group, published in this issue provokes thoughts and provides intellectual inputs in this direction. It is perhaps the first step in the right direction to an unexplored arena, still not fully known to us.
